# Hypothermic Oxygenated Perfusion Versus Static Cold Storage in Liver Transplantation: A Meta‐ Analysis of Randomized Trials

**DOI:** 10.1111/ctr.70676

**Published:** 2026-09-10

**Authors:** Ameni Ellouze, Stylianos Tzedakis, Mohamad Ali Zorkot, Xavier Giudicelli, Karim Boudjema, Heithem Jeddou

**Affiliations:** ^1^ Department of Hepatobiliary and Digestive Surgery University Hospital Rennes 1 University Rennes France; ^2^ Department of Hepato‐biliary, Digestive and Endocrine Surgery Cochin Hospital, APHP, Université Paris Cité Paris France; ^3^ Irset (Institut De Recherche En santé, Environnement Et travail) ‐UMR_S 1085 Inserm, EHESP, Univ Rennes Rennes France

**Keywords:** hypothermic machine perfusion, liver transplantation, static cold storage

## Abstract

**Background:**

Hypothermic oxygenated machine perfusion (HOPE) is an alternative to static cold storage (SCS) for liver graft preservation, aiming to reduce ischemia–reperfusion injury (IRI) and improve post‐transplant outcomes.

**Methods:**

We systematically reviewed randomized controlled trials (RCTs) comparing HOPE with SCS in adult liver transplantation (MEDLINE, EMBASE, CENTRAL to July 2026). Outcomes included early allograft dysfunction (EAD), primary non‐function (PNF), major postoperative complications, biliary complications, including non‐anastomotic strictures (NAS), graft survival, and retransplantation.

**Results:**

Eight RCTs (984 patients; HOPE 461; SCS 523) met inclusion criteria. HOPE significantly reduced EAD (17.8% vs. 33.5%; RR 0.54, 95% CI 0.43–0.68; *p* < 0.00001) and PNF (0.4% vs. 3.6%; RR 0.26, 95% CI 0.09–0.75; *p* = 0.01). HOPE was associated with fewer major complications (Clavien–Dindo ≥ III) (41.2% vs. 50.7%; RR 0.80, 95% CI 0.70–0.93; *p* = 0.003), lower overall biliary complications (18.9% vs. 27.7%; RR 0.72, 95% CI 0.58–0.90; *p* = 0.004) and reduced NAS (3.3% vs. 7.6%; RR 0.43, 95% CI 0.23–0.82; *p* = 0.01). One‐year graft survival was comparable, while retransplantation was reduced after HOPE (RR 0.35, 95% CI 0.14–0.84, *p* = 0.02).

**Conclusion:**

HOPE improves early graft outcomes and reduces biliary morbidity and retransplantation compared with SCS, supporting its use to mitigate IRI.

## 1 Introduction

Liver transplantation (LT) remains limited by the availability of suitable donor organs, prompting the increasing use of extended criteria donor (ECD) liver grafts as a strategy to expand the donor pool [1, 2, 3]. However, ECD grafts are more susceptible to ischemia–reperfusion injury (IRI), which translates into higher rates of adverse post‐transplant outcomes, including early allograft dysfunction (EAD), primary non‐function (PNF), and ischemic cholangiopathy [4, 5]. Static cold storage (SCS) has long been the standard method for liver graft preservation [6, 7]. Although effective and logistically straightforward, SCS provides only limited protection against IRI, particularly in the context of ECD grafts, thereby constraining graft utilization and post‐transplant outcomes [8].

To improve the feasibility and results of LT using ECD grafts, machine perfusion techniques for organ preservation have been increasingly adopted in clinical practice. Among these, hypothermic oxygenated machine perfusion (HOPE), most commonly applied ex situ following an initial period of SCS as an end‐ischemic intervention, has emerged as one of the most widely implemented strategies. Initial clinical evidence, largely derived from observational studies, suggested improved post‐transplant outcomes compared with SCS alone [9, 10, 11, 12, 13, 14]. More recently, several randomized controlled trials (RCTs) have been conducted to provide higher‐level evidence on the clinical impact of HOPE in LT [15, 16, 17, 18, 19, 20, 21, 22].

While multiple systematic reviews and meta‐analyses have evaluated HOPE in LT, most have pooled heterogeneous preservation modalities or combined randomized and non‐randomized studies, limiting the interpretability and strength of their conclusions. To date, no meta‐analysis has focused exclusively on RCTs directly comparing HOPE with SCS [23, 24, 25, 26, 27].

The objective of this study was therefore to perform a comprehensive systematic review and meta‐analysis restricted to currently available RCTs, in order to evaluate post‐transplant outcomes following HOPE versus SCS in LT.

## 2 Materials and Methods

### 2.1 Search Strategy and Study Selection

A systematic literature search was conducted in PubMed, EMBASE, and the Cochrane Library in November 2025, without restriction on publication year. The following search terms were used: (hypothermic oxygenated perfusion OR HOPE OR perfusion OR SCS) AND (LT OR transplant). The complete search strategy is provided in Table S1. Only English‐language studies were included, with no date restrictions.

Two authors (AE and ST) independently screened titles and abstracts. Full texts of potentially eligible studies were reviewed thereafter. Any disagreement was resolved through discussion and arbitration by a third senior author (HJ). When multiple publications reported overlapping data, the most recent study reporting the outcome of interest was retained.

This systematic review and meta‐analysis were conducted in accordance with the Preferred Reporting Items for Systematic Reviews and Meta‐Analyses (PRISMA) guidelines [28]. The study protocol was prospectively registered in the International Prospective Register of Systematic Reviews (PROSPERO) (registration number CRD420261293112)

Only RCTs comparing outcomes after adult LT using grafts preserved with HOPE versus SCS were included.

Studies were excluded if they were retrospective, non‐comparative (case series, case reports, expert opinions, or letters), involved pediatric recipients, split liver grafts, or combined organ transplantation. HOPE was defined as the intervention of interest, and SCS served as the comparator.

### 2.2 Quality Assessment

The quality of the included RCTs was assessed using the Cochrane Collaboration's risk of bias tool [29]. Two investigators (AE and ST) reviewed the publications and assessed the quality independently. Disagreements were resolved by discussion and consensus, confirmed by a senior author (HJ).

### 2.3 Data Extraction

Data were independently extracted by two reviewers (AE and ST) using a structured electronic data extraction form developed according to Cochrane recommendations for intervention reviews. The form was pilot‐tested on a random subset of studies and refined before full application to ensure consistency and completeness.

Extracted data included study characteristics (first author, year of publication, country of origin, journal, study design, and sample size per group), donor and recipient characteristics: donor type (donation after brain death (DBD) or donation after circulatory death (DCD)), donor age and sex, recipient age, recipient Model for End‐Stage Liver Disease (MELD) score, and cold ischemia time (CIT) and post‐transplant outcomes EAD, defined according to the Olthoff criteria [30]; PNF; hepatic artery thrombosis (HAT); overall biliary complications; non‐anastomotic biliary strictures (NAS); anastomotic biliary strictures (AS); severe complications (Clavien–Dindo grade ≥ III) [31]; length of hospital stay; graft loss; and re‐transplantation rates at one year after transplantation).

Any discrepancies were resolved through iterative discussion and consultation with a third independent author (HJ). Complete concordance across all variables was achieved.

### 2.4 Statistical Analysis

For dichotomous outcomes, risk ratios (RRs) with 95% confidence intervals (CIs) were calculated. For continuous variables, mean differences (MDs) were used. When mean values were not available for continuous outcomes, data on median and interquartile range (IQR) were extracted and subsequently converted to mean and standard deviation (SD) using the established equation described by Hozo et al. [32]. When outcomes were reported as percentages only, absolute event numbers were extrapolated.

The individual patient was considered the unit of analysis. Missing outcomes led to exclusion of the corresponding study from the specific analysis. One reviewer (AE) entered the data into Review Manager (RevMan) version 5.4, which was independently checked by two co‐authors (ST and HJ). Statistical analyses were performed in collaboration with an experienced statistician (ST).

A random‐effects model was applied throughout. Results were presented as forest plots with 95% CIs. Statistical heterogeneity was assessed using the Cochran Q test (χ^2^) and quantified using the *I* ^2^ statistic, interpreted as follows: 0%–25% (low heterogeneity), 25%–75% (moderate heterogeneity), and >75% (considerable heterogeneity) [33].

Sensitivity analyses were conducted to evaluate the robustness of the results, including leave‐one‐out analyses in which each study was sequentially excluded. The overall certainty of evidence was assessed using the GRADE framework [34].

## 3 Results

### 3.1 Literature Search and Data Collection

**FIGURE 1:**
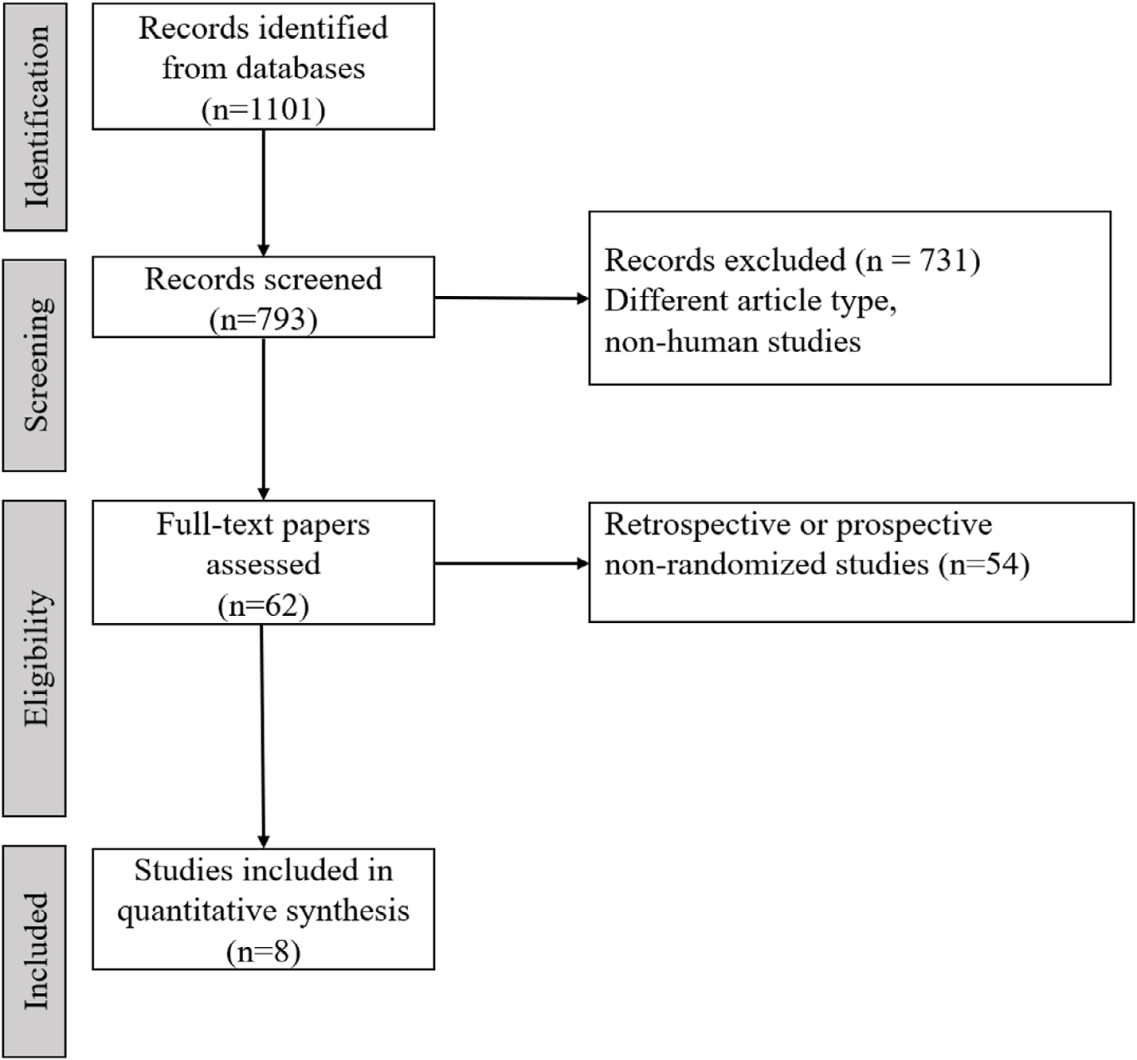
PRISMA flow diagram of study screening and selection.

The initial database search yielded 1101 records, which were screened by title and abstract. Sixty‐two full‐text articles were subsequently assessed for eligibility. Studies that were retrospective or prospective but non‐randomized were excluded. Ultimately, eight RCTs met the inclusion criteria [15, 16, 17, 18, 19, 20, 21, 22] and were retained for quantitative analysis (Figure 1).

These eight trials collectively enrolled 1203 patients, including 570 recipients in the HOPE group and 633 recipients in the SCS group. Two studies included grafts from both donation after brain death (DBD) and donation after circulatory death (DCD) donors [20, 22], whereas five trials included exclusively DBD grafts [16, 17, 18, 19, 21] and one trial included only DCD grafts [15]. In addition, three trials specifically focused on ECD‐DBD livers [16, 17, 21].

HOPE was performed using different perfusion platforms: five trials used the Liver Assist device, two used VitaSmart, and one used the LifePort Liver Transport System.

A summary of baseline characteristics of the study populations and the outcomes of the seven included RCTs is provided in Table 1 and Tables S2, S3.

**TABLE 1:** Characteristics of the eight included RCTs.

Author, year	Centers	No. of centers	Countries	Study period	Number	Perfusion type	Perfusion device & perfusate	CIT before perfusion (h)	Perfusion modality	Perfusion conditions	Perfusion duration (h)	Donor type
**Van Rijn et al., 2021 [** 15 **]**	Multicentre	6	Netherlands Belgium UK	2016–2019	D‐HOPE: 78 SCS: 78	D‐HOPE	Liver Assist Belzer MPS with glutathione	6.2 (5.3–6.9)	Dual	T: 10°C Pressure: PV 5 mmHg, HA 25 mmHg	2.0 (2.0–2.6)	DCD
**Czigany et al., 2021 [** 16 **]**	Multicentre	4	Germany Czech Republic	2017–2020	HOPE: 23 SCS: 23	HOPE	Liver Assist Belzer MPS	6.3 (5.2–7.8)	Single PV	T: 10°C Pressure: PV 3–5 mmHg	2.4 (1.7–3.4)	ECD‐DBD
**Ravaioli et al., 2022 [** 17 **]**	Single	1	Italy	2018–2021	HOPE: 55 SCS: 55	HOPE	Vitasmart Belzer MPS	4.3 (3.6–5.4)	Single PV	T: 10°C Pressure: PV 3 mmHg	2.4 (2.0–3.0)	ECD‐DBD
**Schlegel et al., 2023 [** 18 **]**	Multicentre	10	UK, Belgium, France, Austria, Netherlands, Switzerland	2015–2019	HOPE: 85 SCS: 85	HOPE	Liver Assist Belzer MPS	6.2 (5.0–7.9)	Single PV	T: 8–12°C Pressure: PV 3–4 mmHg; Flow: 150–205 mL/min	1.6 (1.3–2.3)	DBD
**Grat et al** **2023 [** 19 **]**	Single	1	Poland	2021–2022	D‐HOPE : 26 SCS :78	D‐HOPE	Liver Assist Device	NA	Dual	T :12°C Pressure :PV 3‐5mmHg , continuous flow; HA pulsatile flows, systolic pressure 30 mmHg, diastolic pressure 20mmHg	NA	DBD
**Panayotova et al., 2024 [** 20 **]**	Multicentre	9	US	2019–2022	HMP‐O2 : 63 SCS : 73	HMP‐O2	LifePort Liver Transporter and Vasosol preservation solution	4.1 (2.8–5.0)	Dual	Perfusion for 3–7 h at 4°C–8°C Continuos, non pulsatlie, flow‐controlled HA and PV perfusion adjusted by greft weight to 0.66 mL/g liver/minute	2.8 (1.8–3.6)	DBD, DCD
**Lesurtel et al., 2025 [** 21 **]**	Multicentre	8	France	2019–2022	HOPE : 131 SCS :131	HOPE	Liver Assist Belzer MPS IGL‐1	5 .7 (4.7–7)	Single PV	T : 8°C–12°C Pressure : PV 3 mmHg	1–4	ECD‐DBD
**Reich et al., 2026 [** 22 **]**	Multicentre	15	US	2022–2023	HOPE : 109 SCS : 110	HOPE	VitaSmart	4.7 (3.9–5.6)	Single PV	T : 6.7°C Pressure : PV 0.8 mmHg		DBD, DCD

### 3.2 Early Outcomes After LT

#### 3.2.1 EAD

**FIGURE 2:**
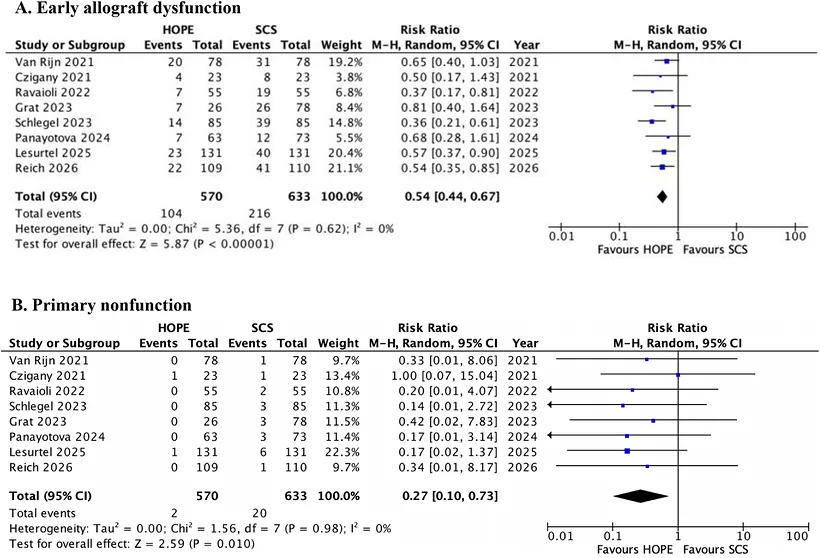
Early outcomes after liver transplantation.

All eight studies reported EAD rates, which occurred in 18.2% (104/570) in the HOPE group compared to 34.1% (216/633) in the SCS group, with low heterogeneity (*I* ^2^ = 0%, *p* = 0.62). HOPE treatment was associated with a significantly lower risk for EAD (risk ratio [RR] 0.54, 95% CI 0.44–0.67, *p* < 0.00001; risk reduction 15.9%) (Figure 2A). EAD was homogeneously defined according to the Olthoff criteria [30] across all eight RCTs, and the certainty of evidence for this outcome was graded as high (Table 2).

**TABLE 2:** Comparison of hypothermic oxygenated perfusion with static cold storage using GRADE approach.

Outcome (studies)	Relative risk / MD (95% CI)	Anticipated absolute effects (95% CI)			Certainty (GRADE)	Interpretation
		SCS	HOPE	Difference		
**Early allograft dysfunction** 8 RCTs) Participants: 1203	RR 0.54 (0.44–0.67)	34.1%	18.4%	15.7% fewer (157 fewer per 1,000; 113 to 191 fewer)	⊕⊕⊕⊕ High	Hypothermic oxygenated perfusion results in a large reduction in early allograft dysfunction.
**Length of hospital stay (days)** (7 RCTs) Participants: 1099	MD −0.48 (−0.87 to −0.08)	Mean 20.0 days	—	−2.04 days	⊕⊕◯◯ Low	Hypothermic oxygenated perfusion may reduce length of hospital stay; results should be interpreted with caution due to substantial heterogeneity.
**Major complications (Clavien–Dindo ≥ IIIb)** (6 RCTs) Participants: 848	RR 0.86 (0.77–0.96)	54.6%	46.7%	7.9% fewer (79 fewer per 1,000; 22 to 126 fewer)	⊕⊕⊕◯ Moderate	Hypothermic oxygenated perfusion likely reduces major postoperative complications.
**Overall biliary complications** (8 RCTs) Participants: 1199	RR 0.72 (0.59–0.89)	27.7%	19.2%	8.5% fewer (85 fewer per 1,000; 31 to 114 fewer)	⊕⊕⊕◯ Moderate	Hypothermic oxygenated perfusion likely reduces overall biliary complications.
**Nonanastomotic strictures** (7 RCTs) Participants:1153	RR 0.50 (0.29–0.88)	7.2%	3.1%	4.1% fewer (41 fewer per 1,000; 9 to 51 fewer)	⊕⊕⊕◯ Moderate	Hypothermic oxygenated perfusion likely reduces non‐anastomotic biliary strictures.
**Graft loss** (7 RCTs) Participants: 974	RR 0.66 (0.42–1.04)	10.8%	6.7%	4.1% fewer (41 fewer per 1,000; 4 more to 63 fewer)	⊕⊕◯◯ Low	Hypothermic oxygenated perfusion may result in little to no difference in graft loss.
**Re‐transplantation** (7 RCTs) Participants: 1047	RR 0.44 (0.22–0.87)	5.6%	2.0%	3.6% fewer (36 fewer per 1,000; 7 to 44 fewer)	⊕⊕⊕◯ Moderate	Hypothermic oxygenated perfusion likely reduces re‐transplantation, although event numbers are limited.

Abbreviations: RCT, randomized controlled trial; RR, risk ratio.

Based on the sensitivity analysis, the removal of individual RCTs by Van Rijn [15] (*p* < 0.00001), Czigany [16] (*p* < 0.00001), Ravaioli [17] (p < 0.00001), Schlegel [18] (*p* < 0.00001), Grat [19] (*p* < 0.00001), Panayotova [20] (*p* < 0.00001), Lesurtel [21] (*p* < 0.00001) or Reich [22] (*p* < 0.00001) did not modify the overall result (Table S4)

#### 3.2.2 PNF

All eight studies reported PNF rates, which occurred in 0.35% (2/570) in the HOPE group compared with 3.2% (20/633) in the SCS group. HOPE was associated with a significantly lower risk of PNF (risk ratio [RR] 0.27, 95% CI 0.10–0.73, *p* = 0.01), with low heterogeneity across studies (*I* ^2^ = 0%, *p* = 0.98) (Figure 2B).

### 3.3 Outcomes Within 1 Year After LT

#### 3.3.1 Major Complications (Clavien–Dindo ≥ III)

**FIGURE 3:**
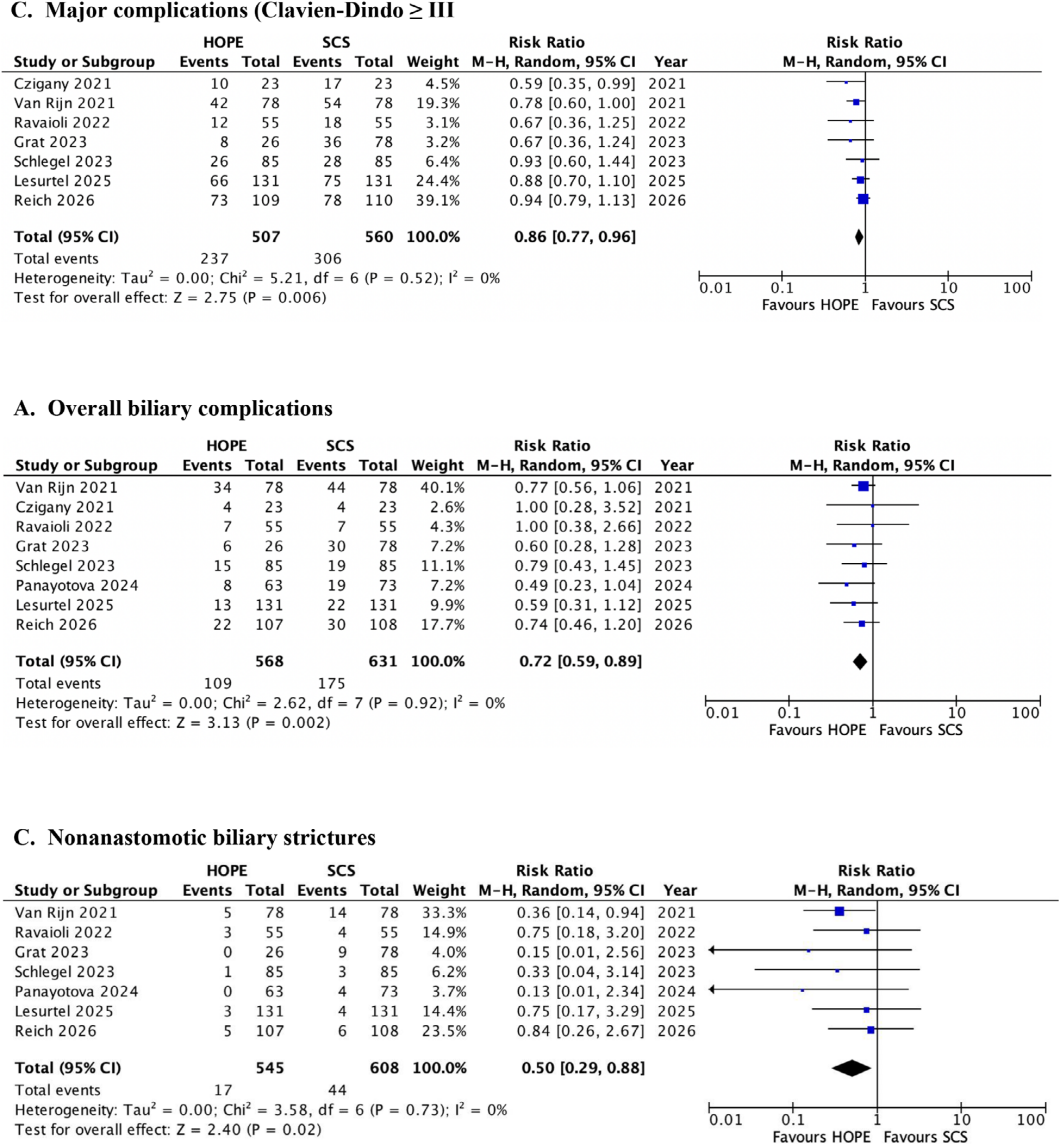
Overall major and biliary complications after liver transplantation.

The incidence of severe complications measured using the Clavien–Dindo classification ≥ III [31] was reported in seven studies [15, 16, 17, 18, 19, 21, 22] and included a total of 1067 patients (HOPE, *n* = **507;** SCS, *n* = 560), with low heterogeneity (*I* ^2^ = 0%, *p* **=** 0.52). HOPE reduced the rate of severe complications compared with SCS (Clavien–Dindo ≥ III; HOPE: 237/507 [46,7%] vs. SCS: 306/560 [54.6%]; risk ratio [RR] 0.86, 95% CI 0.77–0.96, *p* = 0.006) (Figure 3A). The 7.9% reduction in major complications with HOPE was graded as moderate certainty of evidence (Table 2).

### 3.4 Overall Biliary Complications

All eight studies reported overall biliary complication rates, which were higher in the SCS group (*n* = 175/631, 27.7%) compared to the HOPE group (*n* = 109/568 [19.2%]), with low study heterogeneity (*I* ^2^ = 0%, *p* = 0.92). This analysis reached statistical significance (risk ratio [RR] 0.72, 95% CI 0.59–0.89, *p* = 0.002), and the effect was graded as moderate, with a likely 8.5% reduction in overall biliary complications with HOPE (Figure 3B, Table 2).

#### 3.4.1 Non‐anastomotic Biliary Strictures (NAS)

Biliary complications were reported with sufficient detail to allow NAS analysis in seven RCTs [15, 17, 18, 19, 20, 21, 22]. In contrast, Czigany et al. [16] reported an overall biliary complication rate without further details on subgroups. Seven trials [15, 17, 18, 19, 20, 21, 22] were therefore considered to analyze the impact on NAS with a total of 1153 patients (HOPE, *n* = 545, SCS, *n* = 608; low heterogeneity [I2 = 0%, *p* = 0.73]). NAS occurred in 3.1% of patients treated with HOPE (17/545) and in 7.2% of recipients in the SCS group (44/608). HOPE treatment was associated with a NAS risk reduction of 4.1%, graded as a moderate effect (RR 0.50, 95% CI 0.29–0.88, *p* = 0.02) (Figure 3C, Table 2).

### 3.5 Graft Survival

**FIGURE 4:**
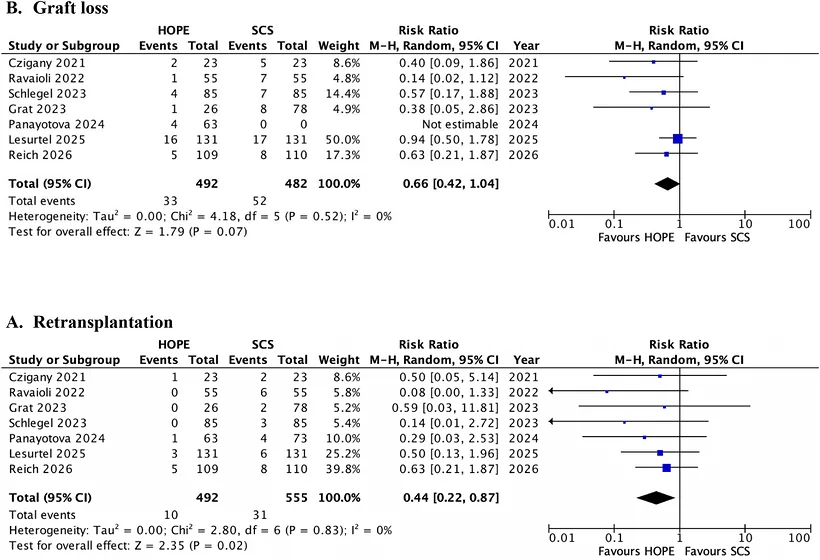
Graft survival and re‐transplantation after liver transplantation.

Graft survival rates after 1‐year follow‐up were pooled from seven RCTs [16, 17, 18, 19, 20, 21, 22]. Graft loss at 1 year was reported for 974 patients (492 HOPE, 482 SCS). In the HOPE group 33 livers (33/492; 6.7%) were lost within 1 year compared to 52 livers in the SCS group (52/482; 10.8%); the heterogeneity was low (I2 = 0%, *p* = 0.52). Although no statistically significant difference was observed between groups (risk ratio [RR] 0.66, 95% CI 0.42–1.04, *p* = 0.07), HOPE was associated with an absolute reduction in graft loss of 4.1%, graded as low certainty of evidence. (Figure 4A, Table 2).

### 3.6 Re‐transplantation

Seven trials [16, 17, 18, 19, 20, 21, 22] reported re‐transplantation rates within 1 year of follow‐up; overall 1047 patients (492 HOPE, 555 SCS) were included. In the HOPE cohort, 10 recipients underwent re‐transplantation (10/492; 2.0%) compared to 31 in the SCS cohort (31/555; 5.6%), which was statistically significant (RR 0.44, 95% CI 0.22–0.87, *p* = 0.02), with a low heterogeneity (I2 = 0%, *p* = 0.83). The absolute risk reduction was 3.6%, graded as moderate certainty (Figure 4B, Table 2).

The detailed analyses of hepatic artery thrombosis, anastomotic biliary strictures, and length of hospital stay are presented in the supplementary material (Figures S1–S3, respectively).

The detailed sensitivity analysis and risk of bias assessment are presented in the supplementary material (Figure S4 and Table S4).

### 3.7 Long Term Outcomes

Only three [15, 16, 19] of the eight included RCTs reported long‐term outcomes beyond the early postoperative period, with follow‐up durations ranging from 2 to 5 years [35, 36, 37]. Owing to heterogeneity in follow‐up length and reported endpoints, long‐term outcomes were synthesized descriptively.

In the randomized trial by Grat et al. [19], long‐term outcomes were reported at 2 years after transplantation in recipients of DBD grafts [35]. There was no significant difference between the HOPE and SCS groups in the overall incidence of biliary complications (23.7% vs. 43.4%, *p* = 0.11), including AS (19.9% vs. 33.7%, *p* = 0.20), NAS (0% vs. 11.1%, *p* = 0.10), and biliary fistulas (11.7% vs. 12.2%, *p* = 0.93). Overall patient survival (92.3% vs. 83.9%, *p* = 0.35) and graft survival (92.3% vs. 81.4%, *p* = 0.23) were comparable between groups. In recipients of high‐risk grafts, graft survival at 2 years was higher in the HOPE group compared with the SCS group (100% vs. 73.1%, *p* = 0.038).

The RCT by Czigany et al. [16] reported long‐term outcomes after a median follow‐up of 48 months [36]. After exclusion of early perioperative morbidity, late morbidity was significantly lower in the HOPE group, with a median reduction of 23 points in the Comprehensive Complication Index (*p* = 0.003) and a lower incidence of major complications (Clavien–Dindo ≥III) compared with SCS (43% vs. 85%, *p* = 0.009). Primary graft loss occurred in 13 patients (HOPE *n* = 3 vs. SCS *n* = 10), resulting in significantly inferior overall graft survival in the SCS group (*p* = 0.029). One‐, three‐, and five‐year graft survival probabilities were 0.913, 0.869, and 0.869 in the HOPE group, compared with 0.783, 0.606, and 0.519 in the SCS group, respectively.

In the RCT by Van Rijn et al. [15], conducted exclusively in DCD LT, long‐term outcomes were reported up to 5 years after transplantation [37]. The incidence of symptomatic NAS was significantly lower in the machine perfusion group than in the control group at 5 years (14% vs. 26%; hazard ratio 0.47, 95% CI 0.23–0.99; *p* = 0.048). New cases of symptomatic NAS occurring beyond the first year were uncommon in both groups. At 5 years, patient survival (63% vs. 70%, *p* = 0.254) and death‐censored graft survival (82% vs. 79%, *p* = 0.806) were comparable between the machine perfusion and SCS groups.

## 4 Discussion

This meta‐analysis is based exclusively on the eight RCTs [15, 16, 17, 18, 19, 20, 21, 22] currently available comparing HOPE with SCS in LT and therefore provides high‐level evidence on the clinical impact of HOPE. Several key findings emerge. First, HOPE led to a significant reduction in EAD compared with SCS, with a large and consistent effect size across all trials and no heterogeneity. This finding was supported by high‐certainty evidence and represents the most robust signal observed in the present analysis. Second, HOPE was associated with a likely reduction in overall biliary complications including NAS, with moderate certainty of evidence. Third, major post‐transplant complications occurred significantly less frequently after HOPE treatment. Finally, re‐transplantation rates within the first year after transplantation were significantly reduced by HOPE compared with SCS, also with moderate certainty of evidence.

In most of the included RCTs, early liver graft injury was the primary endpoint and was assessed using EAD [17, 20, 21, 22] or Model for Early Allograft Function (MEAF) [19] or peak‐alanine aminotransferase levels within 7 days following LT [16].

HOPE significantly reduced EAD rates, an endpoint primarily driven by peak recipient transaminase levels within the first postoperative week [30]. However, the clinical relevance of EAD in the setting of dynamic machine preservation remains uncertain. During HOPE, all grafts release transaminases into the perfusate, which may influence subsequent recipient transaminase levels and thereby confound the assessment of postoperative EAD, reducing its specificity for clinically relevant ischemia–reperfusion injury [10, 16, 38].

In addition, EAD has not been validated as a surrogate marker of long‐term outcomes following machine perfusion [35, 36, 37, 38, 39, 40]. Its association with clinically meaningful endpoints, such as graft survival, ischemic cholangiopathy, and major postoperative complications, remains variable [23, 24, 25]. Consequently, although EAD may reflect early hepatocellular injury, it should not be considered alone. A comprehensive evaluation that includes biliary complications, severe morbidity, and graft survival provides a more reliable assessment of the true clinical impact of preservation strategies.

In this context, HOPE was also associated with a significant reduction in overall biliary complications. AS and NAS are distinct entities with different underlying mechanisms and different post‐transplant clinical courses [15, 41, 42].

In the present meta‐analysis, AS and NAS were evaluated separately. HOPE was associated with a reduction in NAS, whereas no difference was observed for AS. This finding is consistent with the multifactorial determinants of AS, including surgical technique and arterial complications, and with the predominant role of ischemia–reperfusion injury and microvascular damage of the peribiliary plexus in the pathogenesis of NAS [41, 42, 43].

The protective effect of HOPE on NAS is even more relevant, given the strong association between NAS, graft loss, and the need for re‐transplantation. Among the RCTs included, only the trial by Van Rijn et al. [15] was specifically designed to assess NAS as the primary endpoint and demonstrated a significant reduction in symptomatic NAS in DCD graft recipients. Although consistent with large observational studies, further RCTs with NAS as a primary endpoint are warranted.

Another relevant finding of this analysis was the reduction in major postoperative complications associated with HOPE. Although consistent signals were observed in the studies included, interpretation of this outcome was limited by heterogeneity in definitions (Comprehensive Complication Index [16], Clavien–Dindo classification [31]) and reporting timeframes across studies, with complications reported at hospital discharge, 90 days, 6 months, or 1 year. Again, only one randomized trial [18] was adequately powered to assess major post‐transplant complications, while other studies reported serious adverse events without detailed categorization.

Regarding outcomes at one‐year, even in the absence of a statistically significant survival difference, likely reflecting limited power and low event rates rather than absence of effect, the significant reductions in major complications, biliary morbidity, and re‐transplantation support a favorable clinical signal for HOPE.

Unfortunately, long‐term outcomes could not be pooled quantitatively due to heterogeneous follow‐up durations and limited reporting beyond the first postoperative year. Nevertheless, available data particulary in high‐risk ECD grafts suggest that benefits of HOPE tend to persist over time, supporting the concept that the long‐term impact of HOPE is closely linked to its ability to attenuate early ischemia–reperfusion injury [44].

Our meta‐analysis has several limitations. Although the analysis was restricted to randomized trials, the overall number of available RCTs remains limited including diverse donor populations, that is combining both DBD and DCD or extended criteria and standard grafts.

Furthermore, preservation protocols were not uniform across studies. HOPE was performed either as portal vein perfusion alone or as dual hypothermic oxygenated perfusion via both the portal vein and hepatic artery, with additional variability in devices and perfusion duration, potentially influencing the observed effect sizes. Finally, reporting of biliary complications lacked standardization across trials, thereby limiting the precision and granularity of pooled analyses.

In conclusion, this meta‐analysis of RCTs provides high‐level evidence that HOPE is associated with robust reductions in EAD, major complications, biliary morbidity, re‐transplantation, and graft loss. These data reinforce the role of HOPE as a clinically meaningful preservation strategy in LT. Further adequately powered RCTs focusing on long‐term graft survival and biliary outcomes as primary endpoints are needed, particularly in populations restricted to ECD grafts, which are especially vulnerable to ischemia–reperfusion injury.

## Funding

This research did not receive any specific grant from funding agencies in the public, commercial, or not‐for‐profit sectors.

## Conflicts of Interest

The authors declare no conflicts of interest.

## Supporting information




**Supporting File 1**: ctr70676‐sup‐0001‐FigureS1.docx

**Supporting File 2**: ctr70676‐sup‐0002‐FigureS2.docx

**Supporting File 3**: ctr70676‐sup‐0003‐FigureS3.docx

**Supporting File 4**: ctr70676‐sup‐0004‐FigureS4.docx

**Supporting File 5**: ctr70676‐sup‐0005‐TableS1.docx

**Supporting File 6**: ctr70676‐sup‐0006‐TableS2.docx

**Supporting File 7**: ctr70676‐sup‐0007‐TableS3.docx

**Supporting File 8**: ctr70676‐sup‐0008‐TableS4.docx

## Data Availability

The data supporting the findings of this study are available from the corresponding author upon reasonable request.
