# Supplementary material for: Hypothermic Oxygenated Perfusion Versus Static Cold Storage in Liver Transplantation: A Meta‐ Analysis of Randomized Trials
Source: Clin Transplant. 2026 Sep 10;40(9):e70676. doi: 10.1111/ctr.70676 (PMC13559211; doi:10.1111/ctr.70676)
Supplement: Supplementary file 1 [file CTR-40-e70676-s001.docx]

**Table S2 Baseline characteristics of included randomized controlled trials**

| **RCT (first author, year)** | **Donor age (y)** | | **Donor male, n (%)** | | **Donor risk index (points)** | | **Recipient age (y)** | | **Recipient male, n (%)** | | **Recipient lab MELD (points)** | | **CIT before HOPE (min)** | **CIT in SCS (control group) (min)** | **Perfusion time, HOPE (min)** |
| --- | --- | --- | --- | --- | --- | --- | --- | --- | --- | --- | --- | --- | --- | --- | --- |
|  | **HOPE** | **SCS** | **HOPE** | **SCS** | **HOPE** | **SCS** | **HOPE** | **SCS** | **HOPE** | **SCS** | **HOPE** | **SCS** | **HOPE** | **SCS** | **HOPE** |
| **van Rijn et al., 2021** | 52 (43–57) | 49 (37–59) | 52 (67%) | 51 (65%) | 2.12 (1.84–2.38) | 2.12 (1.86–2.42) | 60 (52–65) | 60 (52–65) | 55 (71%) | 52 (67%) | 14 (10–19) | 16 (10–22) | 371 (316–415) | 409 (296–477) | 132 (120–153) |
| **Czigany et al., 2021** | 73 (60–78) | 71 (59–78) | 12 (52%) | 13 (56%) | 2.08 (1.91–2.21) | 1.96 (1.83–2.24) | 60 (52–64) | 63 (56–67) | 18 (78%) | 20 (87%) | 13 (9–18) | 17 (8–25) | 375 (311–465) | 502 (467–584) | 145 (101–203) |
| **Ravaioli et al., 2022** | 76 (64–81) | 72 (59–77) | 31 (56%) | 33 (60%) | 1.846 (1.719–1.908) | 1.766 (1.545–1.908) | 57 (47–65) | 60 (53–66) | 41 (74%) | 39 (71%) | 15 (10–18) | 14 (9–20) | 255 (215–325) | 420 (360–450) | 145 (120–185) |
| **Schlegel et al., 2023** | 59 (48–72) | 62 (44–71) | 45 (53%) | 43 (50%) | NA | NA | 60 (51–64) | 57 (49–64) | 55 (64%) | 67 (79%) | 20 (11–28) | 19 (12–26) | 373 (299–472) | 427 (356–487) | 95.5 (73–137) |
| **Grat et al, 2023** | 53 (40-60) | 44 (35-56) | NA | NA | 1.74 (1.41-1.98) | 1.52 (1.26-1.79) | 46 (39-62) | 51 (41-60) | 18 (69.2%) | 50 (64.1%) | 12 (8-21) | 14 (10-21) | 450 (420-550) | 567 (480-630) | NA |
| **Panayotova et al., 2024** | 48 (36-58) | 38 (30-55) | 40 (63,4%) | 49 (67,1%) | 1.613 (1.397-1.809) | 1.480 (1.225-1.750) | 55 (45-62) | 56 (46-62) | 38 (60.4 %) | 47 (64.4%) | 24 (18, 33) | 22 (14.5, 32) | 384 (324- 438) | 408 (114-468) | 168 (108-216) |
| **Lesurtel et al, 2025** | 68 (57-76) | 70 (55-76) | 77(58.8%) | 72(55.0%) | 1.70 (1.43-1.89) | 1.76 (1.38-1.90) | 60 (53-65) | 60 (54-65) | 106 (80.9 %) | 105 (80.2%) | 17 (11-23) | 14 (9-22) | 316 (263-360) | 388 (313-483) | 112 (83-170) |
| **Reich et al, 2026** | 56.7(47.1-63.6) | 54.4(40.2-62.0) | 61(56.0) | 62(56.4) | 2.1(1.7-2.3) | 2.0(1.7-2.3) | 59.0(49.0-64.0) | 57.5(51.0-65.0) | 68(62.4) | 69(62.7) | 23.0(15.0-26.0) | 21.5(15.0-27.0) | 4.7(3.9-5.6) | 6.1(5.1-6.9) | 121.0(100.5-156.5) |
