# Supplementary material for: Hypothermic Oxygenated Perfusion Versus Static Cold Storage in Liver Transplantation: A Meta‐ Analysis of Randomized Trials
Source: Clin Transplant. 2026 Sep 10;40(9):e70676. doi: 10.1111/ctr.70676 (PMC13559211; doi:10.1111/ctr.70676)
Supplement: Supplementary file 3 [file CTR-40-e70676-s003.docx]

**Table S4. Leave-one-out sensitivity analysis summary**

| **RCT Removed** | **Outcome** | **Heterogeneity (%)** | **Estimate RR** | **Lower bound** | **Upper bound** | **P value** |
| --- | --- | --- | --- | --- | --- | --- |
| Van Rijn et al | EAD | 0 | 0.52 | 0.41 | 0.65 | <0.00001 |
| Czigany et al | EAD | 0 | 0.54 | 0.44 | 0.67 | <0.00001 |
| Ravaioli et al | EAD | 0 | 0.56 | 0.45 | 0.69 | <0.00001 |
| Grat et al | EAD | 0 | 0.52 | 0.42 | 0.65 | <0.00001 |
| Schlegel et al | EAD | 0 | 0.58 | 0.47 | 0.73 | <0.00001 |
| Panayotova et al | EAD | 0 | 0.54 | 0.43 | 0.66 | <0.00001 |
| Lesurtel et al | EAD | 0 | 0.53 | 0.42 | 0.67 | <0.00001 |
| Reich et al | EAD | 0 | 0.54 | 0.43 | 0.68 | <0.00001 |
| Van Rijn et al | PNF | 0 | 0.26 | 0.09 | 0.75 | 0.012 |
| Czigany et al | PNF | 0 | 0.22 | 0.08 | 0.64 | 0.005 |
| Ravaioli et al | PNF | 0 | 0.28 | 0.10 | 0.80 | 0.018 |
| Schlegel et al | PNF | 0 | 0.29 | 0.10 | 0.84 | 0.022 |
| Grat et al | PNF | 0 | 0.25 | 0.09 | 0.73 | 0.011 |
| Panayotova et al | PNF | 0 | 0.29 | 0.10 | 0.82 | 0.02 |
| Lesurtel et al | PNF | 0 | 0.31 | 0.10 | 0.95 | 0.041 |
| Reich et al | PNF | 0 | 0.26 | 0.09 | 0.75 | 0.012 |
| Czigany et al | CD > III | 0 | 0.87 | 0.78 | 0.98 | 0.017 |
| Van Rijn et al | CD > III | 0 | 0.88 | 0.78 | 0.99 | 0.035 |
| Ravaioli et al | CD > III | 0 | 0.86 | 0.77 | 0.97 | 0.01 |
| Grat et al | CD > III | 0 | 0.86 | 0.77 | 0.97 | 0.01 |
| Schlegel et al | CD > III | 0 | 0.85 | 0.76 | 0.96 | 0.006 |
| Lesurtel et al | CD > III | 2 | 0.85 | 0.74 | 0.96 | 0.012 |
| Reich et al | CD > III | 0 | 0.80 | 0.70 | 0.93 | 0.003 |
| Van Rijn et al | OABC | 0 | 0.69 | 0.53 | 0.90 | 0.006 |
| Czigany et al | OABC | 0 | 0.72 | 0.58 | 0.88 | 0.001 |
| Ravaioli et al | OABC | 0 | 0.71 | 0.58 | 0.88 | 0.001 |
| Grat et al | OABC | 0 | 0.73 | 0.60 | 0.91 | 0.004 |
| Schlegel et al | OABC | 0 | 0.72 | 0.58 | 0.89 | 0.002 |
| Panayotova et al | OABC | 0 | 0.75 | 0.61 | 0.92 | 0.006 |
| Lesurtel et al | OABC | 0 | 0.74 | 0.60 | 0.92 | 0.006 |
| Reich et al | OABC | 0 | 0.72 | 0.58 | 0.90 | 0.004 |
| Van Rijn et al | NAS | 0 | 0.60 | 0.30 | 1.19 | 0.14 |
| Ravaioli et al | NAS | 0 | 0.47 | 0.26 | 0.86 | 0.015 |
| Grat et al | NAS | 0 | 0.53 | 0.30 | 0.94 | 0.029 |
| Schlegel et al | NAS | 0 | 0.52 | 0.29 | 0.92 | 0.026 |
| Panayotova et al | NAS | 0 | 0.53 | 0.30 | 0.94 | 0.03 |
| Lesurtel et al | NAS | 0 | 0.47 | 0.26 | 0.86 | 0.015 |
| Reich et al | NAS | 0 | 0.43 | 0.23 | 0.82 | 0.01 |
| Czigany et al | Graft loss | 0 | 0.70 | 0.43 | 1.11 | 0.131 |
| Ravaioli et al | Graft loss | 0 | 0.72 | 0.45 | 1.14 | 0.157 |
| Grat et al | Graft loss | 0 | 0.68 | 0.43 | 1.08 | 0.106 |
| Schlegel et al | Graft loss | 0 | 0.68 | 0.42 | 1.11 | 0.12 |
| Lesurtel et al | Graft loss | 0 | 0.47 | 0.25 | 0.88 | 0.019 |
| Reich et al | Graft loss | 2 | 0.66 | 0.40 | 1.10 | 0.111 |
| Czigany et al | ReLT | 0 | 0.43 | 0.21 | 0.89 | 0.022 |
| Ravaioli et al | ReLT | 0 | 0.49 | 0.24 | 0.99 | 0.047 |
| Grat et al | ReLT | 0 | 0.43 | 0.21 | 0.87 | 0.019 |
| Schlegel et al | ReLT | 0 | 0.47 | 0.23 | 0.95 | 0.035 |
| Panayotova et al | ReLT | 0 | 0.46 | 0.22 | 0.95 | 0.035 |
| Lesurtel et al | ReLT | 0 | 0.42 | 0.19 | 0.93 | 0.032 |
| Reich et al | ReLT | 0 | 0.35 | 0.14 | 0.84 | 0.018 |
| Czigany et al | HAT | 0 | 1.07 | 0.41 | 2.77 | 0.887 |
| Van Rijn et al | HAT | 0 | 0.90 | 0.32 | 2.50 | 0.833 |
| Ravaioli et al | HAT | 0 | 0.83 | 0.32 | 2.13 | 0.693 |
| Grat et al | HAT | 0 | 0.96 | 0.37 | 2.48 | 0.933 |
| Schlegel et al | HAT | 0 | 0.78 | 0.30 | 2.01 | 0.601 |
| Panayotova et al | HAT | 0 | 0.89 | 0.34 | 2.33 | 0.815 |
| Lesurtel et al | HAT | 0 | 1.20 | 0.44 | 3.26 | 0.714 |
| Reich et al | HAT | 0 | 0.80 | 0.30 | 2.14 | 0.661 |
| Van Rijn et al | AS | 0 | 0.79 | 0.56 | 1.12 | 0.182 |
| Ravaioli et al | AS | 0 | 0.86 | 0.65 | 1.15 | 0.319 |
| Schlegel et al | AS | 0 | 0.89 | 0.65 | 1.23 | 0.478 |
| Panayotova et al | AS | 0 | 0.89 | 0.66 | 1.21 | 0.461 |
| Reich et al | AS | 0 | 0.89 | 0.63 | 1.26 | 0.508 |
| Van Rijn et al | LOS | 92 | -0.50 | -0.96 | -0.03 | 0.038 |
| Czigany et al | LOS | 86 | -0.28 | -0.61 | 0.05 | 0.093 |
| Ravaioli et al | LOS | 92 | -0.52 | -0.98 | -0.07 | 0.025 |
| Schlegel et al | LOS | 89 | -0.59 | -1.01 | -0.17 | 0.006 |
| Panayotova et al | LOS | 91 | -0.57 | -1.02 | -0.12 | 0.013 |
| Lesurtel et al | LOS | 91 | -0.55 | -1.04 | -0.06 | 0.027 |
| Reich et al | LOS | 87 | -0.38 | -0.77 | 0.01 | 0.053 |
