# Supplementary material for: Hypothermic Oxygenated Perfusion Versus Static Cold Storage in Liver Transplantation: A Meta‐ Analysis of Randomized Trials
Source: Clin Transplant. 2026 Sep 10;40(9):e70676. doi: 10.1111/ctr.70676 (PMC13559211; doi:10.1111/ctr.70676)
Supplement: Supplementary file 6 [file CTR-40-e70676-s006.docx]

| Table S3. Outcomes of the seven included RCTs | | | | | | | | | | | | | | | | | | | | | | | |
| --- | --- | --- | --- | --- | --- | --- | --- | --- | --- | --- | --- | --- | --- | --- | --- | --- | --- | --- | --- | --- | --- | --- | --- |
|  | | | | | | | | | | | | | | | | | | | | | | | |
| RCT (first author, year) | PNF HOPE n (%) | | EAD HOPE n (%) | | ICU stay HOPE (days) | | LOS HOPE (days) | | Major complications (Clavien ≥III) , n (%) | | Hepatic artery thrombosis, n (%) | | Overall biliary complications, n (%) | | Non-anastomotic biliary strictures, n (%) | | | Anastomotic biliary strictures, n (%) | | Graft loss, n (%) | | Retransplantation, n (%) | |
|  | HOPE | SCS | HOPE | SCS | HOPE | SCS | HOPE | SCS | HOPE | SCS | HOPE | SCS | HOPE | SCS | HOPE | SCS | HOPE | | SCS | HOPE | SCS | HOPE | SCS |
| van Rijn et al., 2021 | 0 | 1 (1) | 20 (26) | 31 (40) | 2 (2–5) | 2 (1–4) | 15 (12–20) | 15 (12–26) | 30 (38.5) | 35 (44.9) | 2 (3) | 2 (3) | 34 (43) | 44 (56.1) | 5 (6) | 14 (18) | 23 (29) | | 22 (28) | NA | NA | NA | NA |
| Czigany et al., 2021 | 1 (4) | 1 (4) | 4 (17) | 8 (35) | 5 (4–8) | 8 (5–18) | 20 (16–27) | 36 (23–62) | 10 (43.5) | 17 (73.9) | 0 | 2 (9) | 4 (17) | 6 (26) | NA | NA | NA | | NA | 2 (8.7) | 5 (21.7) | 1 (4) | 2 (9) |
| Ravaioli et al., 2022 | 0 | 2 (4) | 7 (13) | 19 (35) | 4 (3–8) | 4 (3–6) | 18 (15–28) | 17 (11–41) | 12 (21.8) | 18 (32.7) | 1 (2) | 0 | 7 (13) | 7 (13) | 3 (5) | 4 (7) | 2 (4) | | 2 (4) | 1 (1.8) | 7 (12.7) | 0 | 6 (10.9) |
| Schlegel et al., 2023 | 0 | 3 (3.5) | 14 (16.7) | 39 (45.8) | 3 (2–5) | 3 (2–6) | 17 (12–24.5) | 15 (13–25) | 26 (30.6) | 28 (32.9) | 2 (2.4) | 0 | 15 (17.6) | 19 (22.4) | 1 (1.2) | 3 (3.5) | 14 (16.5) | | 18 (21.2) | 4 (4.7) | 7 (8.2) | 0 | 3 (3.5) |
| Grat et al, 2023 | 0 | 3 (3.8) | 7 (26.9) | 26 (33.3) | 5 (4–6) | 4.5 (4–6) | NA | NA | 8 (30.8) | 36 (46.2) | 0 | 2 (2.56) | 6 (23.7) | 30 (43.4) | 0 (0) | 9 (11.5) | NA | | NA | 1 (3.84) | 8 (10.25) | 0 (0) | 2 (2.56) |
| Panayotova et al., 2024 | 0 (0) | 3 (4.1) | 7 (11.1) | 12 (16.4) | 3 (2-4) | 3 (2-5) | 9 (6-14) | 9 (6-17) | NA | NA | 1 (1.6) | 1 (1.4) | 8 (12.6) | 19 (26) | 0 (0) | 4 (5.5) | 7  (11.1) | | 12 (16.4) | 4 (6.34) | 6 (8.21) | 1 (1.6) | 4 (5.5) |
| Lesurtel et al, 2025 | 1 (0.76) | 6 (4.6) | 23 (17.6) | 40 (30.5) | 6.5 (4-10) | 8 (4-14) | 19 (14-31) | 20 (14-31) | 66 (52.4%) | 75 (61.5) | 1 (0.8) | 4 (3.1) | 13 (9.9) | 22 (16.8) | 3 (2.29) | 4 (3.05) | NA | | NA | 16 (12.2) | 17 (13.0) | 3 (2.3) | 6 (4.6) |
| Reich et al, 2026 | 0 | 1 (0.9) | 22(20.2) | 41(37.3) | 2.0(1.0-4.0) | 2.0(1.0-4.0) | 8.0(6.0-12.0) | 11.0(6.0-15.0) | 73 (66.9%) | 78 (70.9) | 2 (1.9) | 1 (0.9) | 22 (20.6) | 30 (27.8) | 5 (4.7) | 6 (5.5) | 22 (20.6) | | 27 (25.0) | 5 (4.5) | 8 (7.3) | 5 (4.5) | 8 (7.3) |
