# Supplementary material for: Hypothermic Oxygenated Perfusion Versus Static Cold Storage in Liver Transplantation: A Meta‐ Analysis of Randomized Trials
Source: Clin Transplant. 2026 Sep 10;40(9):e70676. doi: 10.1111/ctr.70676 (PMC13559211; doi:10.1111/ctr.70676)
Supplement: Supplementary file 7 [file CTR-40-e70676-s007.docx]

# Table S1. Detailed Search Strategy

| Search No | Search strategy |
| --- | --- |
| #1 | MeSH descriptor: [liver transplant] explode all trees |
| #2 | liver transplant : TI,AB,KW |
| #3 | MeSH descriptor: [liver transplantation] explode all trees |
| #4 | liver transplantation : TI,AB,KW |
| #5 | #1 OR #2 OR #3 OR #4 |
| #6 | MeSH descriptor: [normothermic perfusion] explode all trees |
| #7 | normothermic perfusion : TI,AB,KW |
| #8 | machine perfusion : TI,AB,KW |
| #9 | perfusion : TI,AB,KW |
| #10 | #6 OR #7 OR #8 OR #9 |
| #11 | #5 AND #10 |

This search strategy was applied across the following databases: MEDLINE, EMBASE, and the Cochrane Central Register of Controlled Trials (CENTRAL).
