# Supplementary material for: Hypothermic Oxygenated Perfusion Versus Static Cold Storage in Liver Transplantation: A Meta‐ Analysis of Randomized Trials
Source: Clin Transplant. 2026 Sep 10;40(9):e70676. doi: 10.1111/ctr.70676 (PMC13559211; doi:10.1111/ctr.70676)
Supplement: Supplementary file 11 [file CTR-40-e70676-s008.docx]

**Figure S2 Anastomotic biliary strictures**
